# SARS-CoV-2 infection as a trigger of type 2 diabetes in adults: a population-based cohort study in Sweden using a double negative control design

**DOI:** 10.1007/s10654-026-01422-1

**Published:** 2026-06-26

**Authors:** Josefine Andreasson, Louise Bennet, Jonas Björk, Dominik Dietler

**Affiliations:** 1 Division of Occupational and Environmental Medicine, Lund University, Lund, Sweden; 2 Department of Clinical Sciences in Malmö, Lund University, Lund, Sweden; 3 Clinical Studies Sweden, Forum South, Skåne University Hospital, Lund, Sweden

**Keywords:** COVID-19, SARS-CoV-2, Type 2 diabetes, Test-negative design

## Abstract

Previous studies suggest an increased risk of new-onset type 2 diabetes (T2D) following SARS-CoV-2 infection, which may be subject to detection bias from increased health care contacts among the infected. We aimed to assess the causal effect of SARS-CoV-2 infection on new-onset T2D using a test-negative design and negative control outcomes. We included all individuals aged *≥* 18 years registered in Sweden on 1 February 2020 without prior T2D who ordered a SARS-CoV-2 test through the Swedish healthcare service between 1 February 2020 and 28 February 2022 (*N* = 3,175,958). A test-negative design was applied, matching infected individuals with up to five controls based on birth year, sex, region, vaccination status, and test date. Cox proportional hazards models estimated hazard ratios (HR) and 95% confidence intervals (CI) for the association between infection and new-onset T2D. The influence of COVID-19 severity was assessed using stratified Cox regression. Negative control outcomes anaemia, chronic kidney disease, and thyroid disorder were used to evaluate detection bias. SARS-CoV-2 infection was associated with a 12% (HR 1.12, 95% CI 1.04–1.20) increase in T2D risk, driven by elevated incidence during weeks 1–4 post-infection. Increased risk was concentrated among individuals hospitalized (HR 3.60, 95% CI 2.71-4.78) or admitted to intensive care (HR 4.85, 95% CI 2.60-9.05). Negative control outcomes showed similar patterns. Our findings do not support a causal effect of SARS-CoV-2 infection on new-onset T2D. Instead, observed increases appear largely attributable to increased detection of early-phase, sub-clinical T2D cases during hospitalization or intensive care.

**Supplementary Information:**

The online version contains supplementary material available at https://doi.org/10.1007/s10654-026-01422-1.

## Introduction

Following the emergence of SARS-CoV-2 in December 2019, more than 775 million confirmed cases have been reported globally [1]. During the acute phase of the pandemic, public health initiatives and research largely focused on characterizing the virus, understanding transmission dynamics, and identifying therapeutic targets. More recently, increasing attention has been directed toward the post-acute sequalae of SARS-CoV-2 infection. Prior studies suggest that persistent sequalae encompass a wide range of clinical manifestations and that the associated risk and burden increase with the severity of COVID-19 illness [2, 3].

One proposed sequela of SARS-CoV-2 infection is the development of type 2 diabetes (T2D), a chronic condition associated with complications such as retinopathy, chronic kidney disease, and neurological impairment [4], as well as increased cardiovascular morbidity and mortality [5]. Meta-analyses have reported a 30–47% increased risk of T2D following SARS-CoV-2 infection, with the risk of new-onset T2D increasing with COVID-19 severity [6, 7], while vaccination status at the time of infection appears to be protective [8, 9]. Notably, this association has not yet been investigated in the Swedish population. Several mechanisms have been proposed to explain the increased risk of new-onset T2D following SARS-CoV-2 infection, including virus-induced pancreatic injury, systemic inflammation leading to insulin resistance, and glucocorticoid-related hyperglycaemia [10].

However, the existing literature examining the association between SARS-CoV-2 infection and new-onset T2D may be affected by several sources of bias. First, selection bias may arise due to selective testing practices or differences in individuals’ testing behaviour. Second, detection bias may occur if individuals who seek care for COVID-19 have an increased likelihood of receiving a T2D diagnosis during these encounters. Previous studies comparing the risk of new-onset diabetes among hospitalized and non-hospitalized COVID-19 patients have either not incorporated negative control outcomes [9, 11–18] or have relied on suboptimal negative control outcomes involving conditions that are not routinely evaluated during COVID-19-related hospitalizations [8, 19, 20].

The primary aim of this study was to assess whether adults infected with SARS-CoV-2 develop T2D at a higher rate than comparable test-negative individuals and to characterize the time-varying risk following infection. By using a double-negative control design including nationwide registers, we explore the potential contribution of detection and selection bias to strengthen causal inference. In a series of sensitivity analyses, we evaluate whether associations varied by source of T2D diagnose data or COVID-19 severity.

## Methods

### Study design and study population

The source population for this study consists of all individuals *≥* 18 years residing in Sweden on February 1 st, 2020, that ordered a SARS-CoV-2 test through the Swedish healthcare service “1177” at any time between February 1 st, 2020, and February 28th, 2022, without a previous record of T2D. We created a matched test-negative cohort in which COVID-19 cases tested through “1177” were matched with up to 5 controls who tested negative within 7 days before or after the index date. Controls were matched on sex, birth year, region of residence, and vaccination status (< 2 vs. ≥2 doses 14 days before the index date). Controls were ineligible if they had a COVID-19 diagnosis prior to their negative test. In addition, all individuals having a diabetes diagnosis (ICD-10 E08-E14) or prescription of glucose lowering medication indicating diabetes (ATC A10A/A10B) prior to index date were excluded. Matching was performed with replacement, allowing an unexposed control to be matched to multiple exposed individuals provided they met the matching criteria. Individuals who tested positive for COVID-19 were considered exposed from the date of their positive test and became eligible to be matched to unexposed controls. The matched cohort participants were followed from index date until new-onset T2D, or the earliest occurrence of SARS-CoV-2 infection, other diabetes diagnosis, emigration, death, or end of follow-up (February 28th, 2022).

### Data sources

Data were obtained from multiple national registers with complete population coverage linked using the personal identification number [21]. All data were pseudonymized before disclosure. Data on ordered COVID-19 tests were obtained from the national website and helpline for healthcare information, advice, and e-services "1177" managed by Inera. Data from “1177” were available for all Swedish regions except Region Östergötland. Data on all positive laboratory-confirmed SARS-CoV-2 tests were obtained from SmiNet held by the Public Health Agency of Sweden which also provided data on COVID-19 vaccinations through the National Vaccination Register. Statistics Sweden provided socio-demographic and socio-economic information, including migration, death, family linkage, education, and income.

For the main analyses using national-level data, information on T2D diagnoses was sourced from the Swedish National Diabetes Register (NDR, available between 2010 and 2022) maintained by the Västra Götaland Region. NDR is a nationwide clinical quality register covering approximately 88% of individuals with diabetes [22]. Information on the year of onset was available before 2010 given that individuals had a health care visit in subsequent years. As NDR only reports the year of disease onset, the exact date of diagnosis was sourced from the National Patient Register (including both inpatient care and specialized outpatient care) from the National Board of Health and Welfare (Socialstyrelsen; available between 2005 and 2023). In addition, the expedition date of anti-diabetic medication was taken from the National Prescribed Drug Register (Socialstyrelsen), which contains information on all prescribed drugs dispensed at pharmacies between 2005 and 2023. Admissions to an intensive care unit (ICU) for COVID-19 was sourced from the Swedish Intensive Care Registry, a national quality register for intensive care.

For additional analyses, we used the dates of diagnoses made in primary care to determine the date of T2D onset. Primary care data were available from the three largest Swedish healthcare regions, Region Stockholm (available for 2015–2023), Västra Götaland Region (available for 2015–2023), and Scania Region (available for 2016–2023), representing around 54% of all Swedish residents [23].

### Variables

The primary exposure was real-time reverse transcription polymerase chain reaction (RT-PCR)-confirmed SARS-CoV-2 infection, identified through data from “1177”. Tests with a positive diagnostic for SARS-CoV-2, missing diagnostic, or technical error, for which a confirmed infection was registered in SmiNet within 7 days before or after the test date, was identified as positive and the test date closest to the date of registration in SmiNet was selected as index date. The date of the first positive test was used for individuals with multiple positive tests. Positive tests were excluded if they were not registered in the national reporting system for notifiable infectious diseases (SmiNet) within 7 days of the test date or if the individual had a prior COVID-19 diagnosis. Individuals with a negative test were considered as controls.

**Fig. 1:**
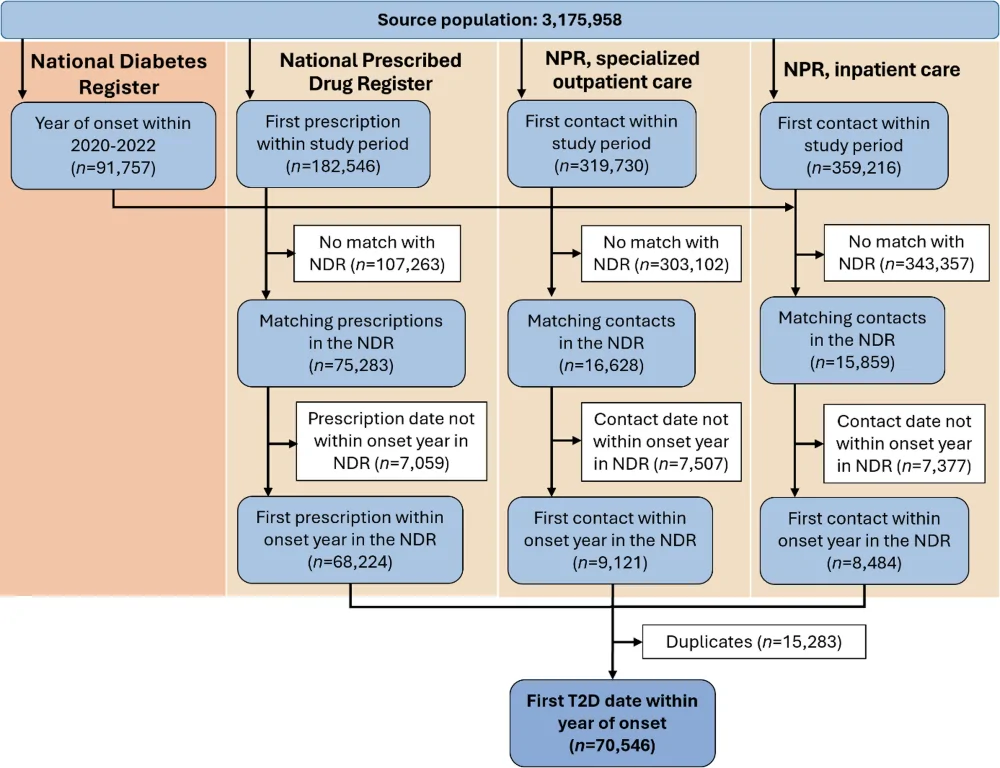
Data extraction from national registers to identify date of new-onset type 2 diabetes within the study period February 1 st, 2020-December 31 st, 2022. NDR; National Diabetes Register, NPR; National Patient Register, T2D; type 2 diabetes

The outcome of interest was time until new-onset T2D. New-onset T2D was defined as a new entry in the NDR with a first-time record of either a T2D diagnosis or an expedition of an anti-diabetic medication within the year of onset in the NDR. We triangulated data from the NDR, the National Patient Register, and the National Prescribed Drug Register to derive the date of T2D onset. The earliest of a T2D or unspecified diabetes diagnosis (ICD-10 codes E11 and E14) or expedition date of anti-diabetic medication (ATC A10A/A10B) served as potential onset date of T2D. Since the date of symptom onset is crucial for this analysis, we excluded individuals whose earliest T2D onset date in the National Patient Register or the National Prescribed Drug Register did not fall within the year of onset reported in the NDR (Fig. 1). Individuals had to be registered in Sweden for at least 1 year before T2D onset date to capture pre-existing T2D. The final number of T2D cases with a reliable onset date comprised 77% (70,546/91,757) of all new cases registered in the NDR during the inclusion period 2020–2022.

In addition to the matching variables (age, sex, region of residence, test date, and vaccination status), we considered the following covariates: “Migration status” was defined as “native born” if born in Sweden and both parents born in Sweden, “second generation immigrant” if born in Sweden, with one or both parents born abroad, and “first generation immigrant” if born abroad. “Non-Western origin” among first generation migrants was defined as being born in a country in the Middle East, Africa, Asia, South America, former Soviet Union, or if registered as nationless. “Western origin” was defined as being born in the remaining regions except Sweden [24]. “Individual disposable income” was categorized into quintiles of “lowest”, “low”, “middle”, “high” or “highest”. “Educational attainment” was categorized as “primary”, “upper secondary, 2 years”, “upper secondary, 3 years”, “Tertiary, < 3 years”, and “Tertiary, *≥* 3”. Missing disposable income (*N* = 7,464, 0.2%) and educational attainment (*N* = 17,775, 0.6%) were assigned the median value and mode for the total population, respectively. “Parental history of diabetes” was defined as any biological parent having any diabetes diagnosis in the National Patient Register or having been prescribed anti-diabetic drugs in the National Prescribed Drug Register prior to index date. We also used prescribed drugs expedited within 2 years prior to index date that are potentially being associated with the outcome and survival as covariate, including lipid-lowering drugs and antihypertensive drugs (ATC C02, C07-C10). Comorbidities based on diagnoses during or before the month preceding the index included hypertension (ICD-10 I10-I15), atherosclerotic cardiovascular disease (ICD-10 I25), chronic obstructive lung disease (ICD-10 J44), heart failure (ICD-10 I50), atherosclerosis (ICD-10 I70), tumours and cancers (ICD-10 C00-D48), and obesity (ICD-10 E66).

### Statistical analysis

The association between SARS-CoV-2 infection and time until T2D onset was assessed using Cox proportional hazards regression. The first day following index date was excluded to avoid reverse causality. Models were adjusted for highest attained education, individual disposable income, migration status, parental history of diabetes, number of comorbidities, and previously prescribed drugs. Analyses were further stratified by time since infection; first four weeks (acute phase), 5–28 weeks (early post-acute phase), 29–52 weeks (late post-acute phase), and 53–102 weeks (long-term follow-up). To account for individual-level clustering due to the matching with replacements, robust variances were calculated using the Huber sandwich estimator in all Cox regression models [25]. Results were presented as hazard ratio (HR) and corresponding 95% confidence intervals (CI).

### Sensitivity analyses

In Sweden, T2D in adults is primarily diagnosed by physicians in primary health care settings, with particularly complex cases referred to hospital or specialist outpatient settings [26]. Hence, many T2D diagnoses are made in primary care settings. To account for this, a sensitivity analysis was conducted integrating diagnoses made in primary care in a cohort of individuals *≥* 18 years residing in Region Stockholm, Västra Götaland Region, and Scania Region on February 1 st, 2020. The same strategy to identify T2D onset was used with the extension of using primary care diagnoses (Supplementary Information [SI] Fig. 1).

To evaluate the risk of new-onset T2D by COVID-19 severity, we performed sensitivity analyses in which the effect of SARS-CoV-2 infection was modelled as a time-dependent exposure stratified by disease severity. This approach allowed individuals to transition to higher categories of COVID-19 severity over time while preserving the original matched sets. Severity was classified as hospitalization or admission to an intensive care unit. In sub-analyses restricted to regions with available primary care data, primary care contact was included as an additional category. All healthcare encounters were required to have COVID-19 (ICD-10 U07.1/U07.2) recorded as main diagnosis. Follow-up time in these analyses spanned from the earliest date at a given severity level (e.g., hospital admission) until either reaching a more severe severity level (e.g., ICU admission) or any of the other end points outlined above.

### Negative control outcome analyses

In additional analyses, we used negative control outcomes to investigate whether the association between SARS-CoV-2 infection and new-onset T2D among individuals seeking health care with COVID-19 was influenced by detection through routine testing of sub-clinical T2D cases during healthcare contacts. We included negative control outcomes that are not causally linked to COVID-19 infections but may be coincidentally detected during healthcare visits similar to T2D. Negative control outcomes included anaemia (ICD-10 D50-D64), chronic kidney disease (ICD-10 N18), and disorders of the thyroid gland (ICD-10 E00-E07) identified in the National Patient Register. Disease onset was defined as the earliest date registered with respective ICD-10 code. The same modelling strategy was used to estimate the effect of SARS-CoV-2 infections on the hazard to be diagnosed with each negative controls as outcome of interest.

## Results

Between February 1 st, 2020, and February 28th, 2022, we identified 3,175,958 individuals aged 18 years or older without prior T2D diagnosis that ordered a SARS-CoV-2 test ordered through “1177”. Of these, 936,119 were individuals with a first positive test and 2,448,858 individuals with a total of 5,774,779 negative tests. After excluding individuals according to the predefined criteria, the final study population comprised 921,835 exposed individuals matched to 4,443,379 unexposed observations. Table 1 summarizes the characteristics of the study population.

**Table 1:** Characteristics of exposed individuals (tested positive) and unexposed observations (tested negative) for SARS-CoV-2 during the study period (February 1 st, 2020-February 28th, 2022), matched on sex, age, vaccination status, region, and test date

	Exposed	Unexposed
n	921,835	4,443,379
Female	485,219 (52.6)	2,361,526 (53.1)
Age group^a^		
18–64 years	884,807 (96.0)	4,289,381 (96.5)
65 years or older	37,028 (4.0)	153,998 (3.5)
Vaccinated (≥ 2 doses)^a^	413,643 (44.9)	2,252,441 (50.7)
Migration status^b^		
Native born	607,115 (65.9)	3,132,735 (70.5)
Second generation immigrant	120,982 (13.1)	585,375 (13.2)
First generation immigrant, Western origin	78,781 (8.5)	323,962 (7.3)
First generation immigrant, non-Western origin	114,854 (12.5)	401,006 (9.0)
Individual disposable income (SEK)	3,101 (2,296- 4,057)	3,107 (2,355- 4,064)
Highest attained education		
Primary	71,083 (7.7)	281,559 (6.3)
Upper secondary, 2 years	130,118 (14.1)	582,154 (13.1)
Upper secondary, 3 years	269,008 (29.2)	1,265,917 (28.5)
Tertiary, < 3 years	154,551 (16.8)	715,041 (16.1)
Tertiary, *≥* 3 years	297,075 (32.2)	1,598,708 (36.0)
Number of comorbidities^c^		
0	749,788 (81.3)	3,544,714 (79.8)
1	155,238 (16.8)	807,358 (18.2)
2	14,351 (1.6)	78,633 (1.8)
≥ 3	2,458 (0.3)	12,674 (0.3)
Prescribed drugs^d^	109,926 (11.9)	576,825 (13.0)
Parental history of diabetes	215,047 (23.3)	1,077,023 (24.2)

Values are *n* (%) for categorical variables and median (interquartile range) for continuous variables. SEK; Swedish Krona

^a^at start of follow-up

^b^Missing in 103 exposed and 301 unexposed individuals

^c^Includes hypertension, atherosclerotic cardiovascular disease, chronic obstructive lung disease, heart failure, atherosclerosis, tumours and cancers, and obesity

^d^Includes lipid-lowering and antihypertensive drugs

**Fig. 2:**
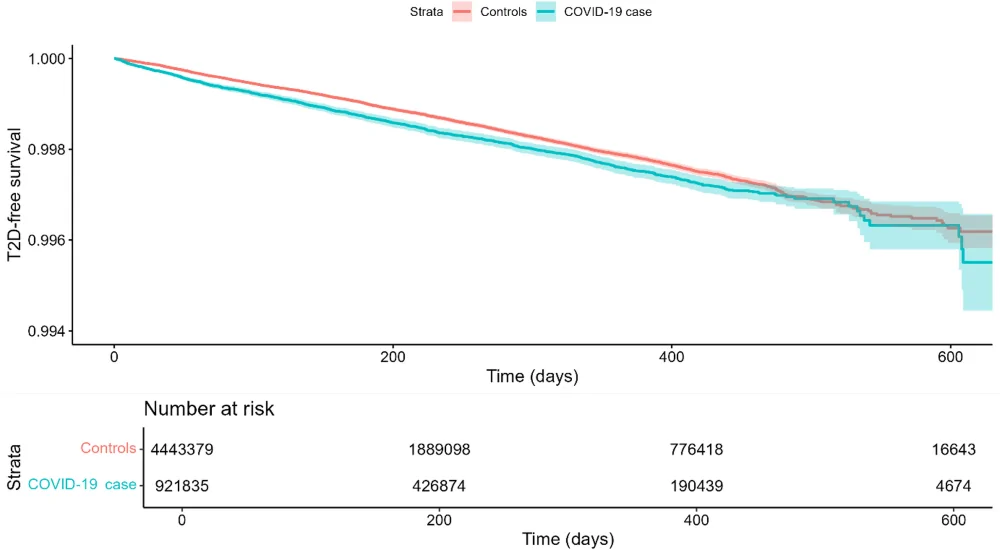
Kaplan-Meier curves comparing COVID-19 exposed with controls. Data was retrieved from nationwide registers. Individuals are followed from one day post SARS-CoV-2 infection until type 2 diabetes (T2D), emigration, death, or the end of the study period (28 February 2022)

**Fig. 3:**
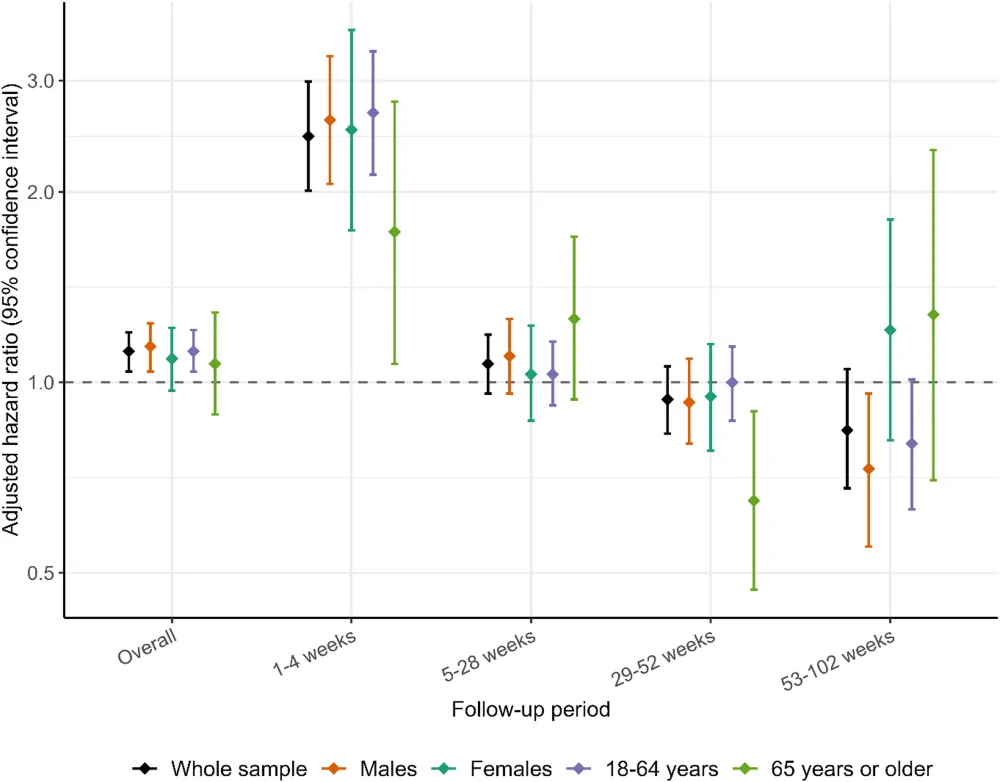
Adjusted hazard ratios (HR) with 95% confidence intervals (95% CI) for the rate of developing type 2 diabetes in a cohort of individuals with confirmed SARS-CoV-2 infection and up to five control individuals matched by sex, age, geographical region, vaccination status, and test date, in the total population and stratified by sex and age. The categories for the follow-up period correspond to time since infection. HR and 95% CI were derived using Cox regression adjusting for migration status, number of comorbidities, prior prescriptions, individual disposable income, highest attained education, and parental diabetes

During a median follow-up of 89 days (IQR 40–368) among exposed individuals and 62 days (IQR 23–347) among non-exposed individuals, we identified 1,273 and 4,687 new-onset T2D cases, respectively. Crude T2D incidence rate per 100,000 person-years was higher among exposed individuals (IR 247, 95% CI 234–261) than among their unexposed counterparts (IR 212, 95% CI 206–219). Kaplan-Meier curves demonstrated an increased cumulative incidence of T2D among exposed individuals (Fig. 2). Across the entire study period, exposed individuals had a 12% higher adjusted hazard of new-onset T2D compared with matched unexposed (HR 1.12, 95% CI 1.04–1.20, Fig. 3; see Supplementary Table 1 for full model results). Stratified analyses showed that the risk of T2D among exposed individuals was only elevated during the first four weeks following SARS-CoV-2 test date (HR 2.45, 95% CI 2.01–2.99). In contrast, no differences in hazard ratios were observed 5–28 weeks (HR 1.07, 95% CI 0.96–1.19), 29–52 weeks (HR 0.94, 95% CI 0.83–1.06), or 53–102 weeks (HR 0.84, 95% CI 0.68–1.05) following test date (Fig. 3). Males and females, as well as individuals 18–64 years and 65 years or older, exhibited comparable hazard patterns across all follow-up periods.

In sensitivity analyses extending the T2D case definition to also include T2D diagnoses made in primary care (Supplementary Fig. 1), an additional 5,492 individuals with new-onset T2D were identified and 6,688 individuals received an earlier date of onset. Analyses in this sub-cohort with T2D diagnoses available from primary care registers showed similar temporal patterns but no increased hazard over the entire study period (Supplementary Fig. 2, Supplementary Fig. 3). Compared with their unexposed counterparts, exposed individuals had a hazard ratio for new-onset T2D of 1.07 (95% CI 0.95–1.21) over the entire study period. The HR was 2.67 (95% CI 1.88–3.78) in weeks 1–4, 0.98 (95% CI 0.82–1.17) in weeks 5–28, 1.00 (95% CI 0.82–1.22) in weeks 29–52, and 0.73 (95% CI 0.51–1.03) in weeks 53–102.

**Fig. 4:**
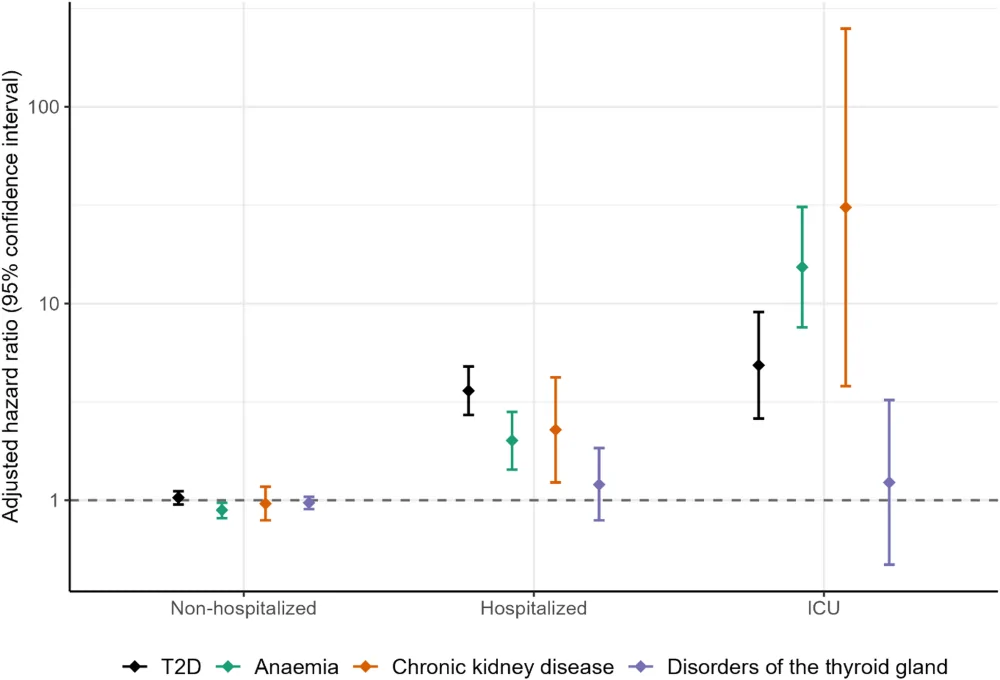
Adjusted hazard ratios (HR) with 95% confidence intervals (95% CI) for the rate of developing type 2 diabetes (T2D), anaemia, chronic kidney disease, or disorders of the thyroid gland in a cohort of individuals with confirmed SARS-CoV-2 infection and up to five control individuals matched by sex, age, geographical region, vaccination status, and test date, stratified by COVID-19 severity. Data was retrieved from nationwide registers. HR and 95% CI were derived using Cox regression adjusting for migration status, number of comorbidities, prior prescriptions, individual disposable income, highest attained education. The T2D model was additionally adjusted for parental diabetes

Kaplan-Meier curves stratified by COVID-19 severity demonstrated a higher cumulative incidence of T2D among individuals who were hospitalized or admitted to the ICU, with the increased risk emerging shortly after hospitalization or ICU admission (Supplementary Fig. 4). In adjusted Cox regression models, individuals who were not hospitalized during follow-up did not exhibit an elevated risk of new-onset T2D compared with unexposed individuals (HR 1.03, 95% CI 0.95–1.11). However, the risk of new-onset T2D was markedly higher during periods of hospitalization (HR 3.60, 95% CI 2.71–4.78) and ICU admission (HR 4.85, 95% CI 2.60–9.05) (Fig. 4). Interestingly, sub-group analyses among residents in the regions with available primary care data indicated no difference in new-onset T2D during periods in which individuals sought primary care (HR 0.98, 95% CI 0.76–1.25). Findings for the non-hospitalized (HR 0.98, 95% CI 0.85–1.12), hospitalized (HR 3.53, 95% CI 2.23–5.58), and ICU-admitted groups (HR 11.55, 95% CI 4.06–32.82) aligned with the main results (Supplementary Fig. 5, Supplementary Fig. 6).

To assess the potential influence of detection bias among exposed individuals engaging with health care services, survival analyses were performed using negative control outcomes. Associations between COVID-19 hospitalization and anaemia and chronic kidney disease diagnoses were comparable to the estimates found for the association with T2D. Among individuals admitted to the ICU, associations between a positive COVID-19 test and anaemia and chronic kidney disease respectively, were even stronger than for T2D (Fig. 4, Supplementary Fig. 7a-c). No elevated risk of thyroid disorders was observed in any exposure group.

## Discussion

In this matched cohort study using a double negative control design, we found that the elevated risk of new-onset T2D following SARS-CoV-2 infection was largely confined to the first four weeks after a positive test. This early increase was predominantly observed among individuals with severe disease, defined as hospitalization or ICU admission for COVID-19. Moreover, negative control outcomes exhibited similar short-term elevations and subsequent attenuation, indicating that previously reported post-COVID-19 estimates may be substantially influenced by detection through routine testing for T2D during healthcare contact rather than a sustained causal effect of the infection. To our knowledge, this is the first study to apply a matched, double negative control strategy leveraging nationwide registers with complete population coverage to address selection and detection biases in the association between SARS-CoV-2 infection and new-onset T2D.

In line with prior reports [8, 9, 11–14, 17, 18, 20], we observed an increased risk of new-onset T2D among individuals who tested positive for SARS-CoV-2 compared to their test-negative controls during the overall study period. However, when stratifying by time since the test date, we found that this elevated risk was largely restricted to the first four weeks after testing. This pattern aligns with findings by Taylor et al., who reported a markedly increased risk of T2D during the first four weeks following a COVID-19 diagnosis, particularly among hospitalized patients [27].

T2D is commonly preceded by a prolonged subclinical phase. In a recent Swedish population-based study, nearly half of individuals who developed T2D remained undiagnosed over a ten-year period, particularly among younger individuals [28]. Because blood glucose levels and related laboratory measures are routinely assessed during hospital care, the early post-infection rise in recorded T2D may partly reflect earlier detection of pre-existing but previously undiagnosed disease. Reges et al., observed a pronounced separation in cumulative hazard of new-onset diabetes among patients hospitalized with COVID-19 compared with non-COVID-19 controls, with no corresponding difference observed among non-hospitalized individuals [15]. More broadly, several studies have described a dose-response relationship between COVID-19 severity and subsequent T2D risk [13, 16, 19, 20, 29]. Notably, previous studies have suggested that diabetes incidence after hospitalization for COVID-19 is similar to that after hospitalization for pneumonia [30], indicating that acute illness and healthcare contact rather than a SARS-CoV-2-specific effect may partly drive the association.

While several prior studies sought to reduce detection bias linked to COVID-19-related hospitalization using landmark designs [15] or sample restrictions [12], only one incorporated sensitivity analyses with negative control outcomes based on diagnostic code and laboratory-based outcomes [20], reporting elevated post-acute diabetes risk even among non-hospitalized individuals and graded increases with care intensity. Importantly, no association were observed for the negative control outcomes [20]. In contrast, our negative control outcomes were selected to closely resemble T2D with respect to clinical detection and diagnostic pathways, are routinely assessed at hospital admission, and are not known sequalae of SARS-CoV-2 infection. The fact that these negative controls mirrored the early spike seen for T2D supports detection as a key driver of the initial elevation.

A major strength of this study is the combined use of a test-negative design and negative control outcomes to reduce and quantify bias. In Sweden, progressively standardized COVID-19 testing protocols were implemented from spring 2020, which produced high-quality, population-wide testing data. These data enabled precise matching of individuals with confirmed SARS-CoV-2 infection to test-negative comparators within narrow time windows, thereby mitigating confounding from health-seeking behaviour and access to testing. Some exposure misclassification may have occurred if individuals were infected prior to their first recorded positive test because of non-testing or false-negative results. However, this misclassification is likely non-differential with respect to T2D and would therefore be expected to bias estimates toward the null. In addition, older individuals including nursing home residents, may have been tested preferentially in a healthcare setting rather than through “1177”, potentially contributing to the underrepresentation of older individuals in the study population.

Another strength is the use of high-quality, extensive Swedish register data. By linking the national quality register NDR with several national public authority registers and supplementing these with primary care data from the three largest regions, we could accurately determine the date of first T2D diagnosis. Given that contemporary treatment guidelines for conditions such as heart failure and chronic kidney disease increasingly include glucose-lowering medications, extending their use beyond patients with diabetes [31, 32], prescription data alone has become less reliable source for identifying new-onset T2D. By restricting new-onset T2D case identification to individuals registered in the NDR, we were unable to capture approximately 12% of all diabetes cases that are not represented in the register. Furthermore, only 77% of NDR-registered cases were included in the analytical sample, as case dates could not be assessed accurately. Consequently, roughly one third of new-onset T2D cases in Sweden may not have been included in the present study, which should be considered when interpreting the findings. Yet, when some of these missed cases were recuperated using primary care data, results were robust. Any remaining outcome misclassification is likely to be non-differential and would be expected to bias results toward the null.

Overall, our results suggest that the observed increase in T2D incidence after SARS-CoV-2 infection is primarily driven by increased detection among individuals receiving hospital or ICU care, rather than a persistent SARS-CoV-2 effect. Replication of this double negative control approach in other settings would help clarify the relationship between COVID-19 and new-onset T2D and to further quantify the contribution of detection bias.

## Supplementary Information

Below is the link to the electronic supplementary material.

Supplementary Material 1

## Data Availability

The individual-level data used in this paper was retrieved from a variety of Swedish registers kept by public agencies such as Statistics Sweden and the National Board of Health and Welfare, and the Centre of Registers Västra Götaland. The data are classified and not publicly available due to data privacy laws. Access to the data requires approval from Stockholm University and SWECOV following ethical review.
