# Supplementary material for: SARS-CoV-2 infection as a trigger of type 2 diabetes in adults: a population-based cohort study in Sweden using a double negative control design
Source: Eur J Epidemiol. 2026 Jun 26;41(8):959–67. doi: 10.1007/s10654-026-01422-1 (PMC13612641; doi:10.1007/s10654-026-01422-1)
Supplement: Supplementary file 1 [file 10654_2026_1422_MOESM1_ESM.docx]

**Supplementary information**

**SARS-CoV-2 Infection as a Trigger of Type 2 Diabetes in Adults: a Population-Based Cohort Study in Sweden Using a Double Negative Control Design**

**European Journal of Epidemiology**

Josefine Andreasson^1^*, Louise Bennet^3^, Jonas Björk^1,2^, Dominik Dietler^1^

^1^ Division of Occupational and Environmental Medicine, Lund University, Lund, Sweden

^2^ Clinical Studies Sweden, Forum South, Skåne University Hospital, Lund, Sweden

^3^ Department of Clinical Sciences in Malmö, Lund University, Lund, Sweden

* Corresponding author: Josefine Andreasson ([josefine.andreasson@med.lu.se](mailto:josefine.andreasson@med.lu.se) )

| **Supplementary Table 1** | Estimates of the main Cox regression model |
| --- | --- |
| **Supplementary Figure 1** | Data extraction of type 2 diabetes from primary care registers |
| **Supplementary Figure 2** | Kaplan-Meier survival curves for sub-cohort including primary care data |
| **Supplementary Figure 3** | Cox regression model for sub-cohort including primary care data |
| **Supplementary Figure 4** | Kaplan-Meier survival curves by COVID-19 severity |
| **Supplementary Figure 5** | Kaplan-Meier survival curves for sub-cohort including primary care data by COVID-19 severity |
| **Supplementary Figure 6** | Cox regression model for sub-cohort including primary care data by COVID-19 severity |
| **Supplementary Figure 7** | Kaplan-Meier survival curves for the negative outcomes anaemia, chronic kidney disease, and disorders of the thyroid gland, by COVID-19 severity |

**Supplementary Table 1** Estimates of the main Cox Proportional Hazards regression model for the total study population

| **Variable** | **HR** | **Lower 95% CL** | **Higher 95% CL** | ***p*** |
| --- | --- | --- | --- | --- |
| **SARS-CoV-2 infection** | 1.12 | 1.04 | 1.20 | 0.003 |
| **Migration status** |  |  |  |  |
| Native born | Ref. | Ref. | Ref. | Ref. |
| Second generation | 1.11 | 0.97 | 1.27 | 0.118 |
| Western | 1.37 | 1.19 | 1.58 | <0.001 |
| Non-western | 3.26 | 2.88 | 3.68 | <0.001 |
| **Individual disposable income** | 1.00 | 1.00 | 1.00 | 0.021 |
| **Educational level** |  |  |  |  |
| Primary | 1.86 | 1.60 | 2.16 | <0.001 |
| Upper secondary, 2 years | 1.60 | 1.40 | 1.84 | <0.001 |
| Upper secondary, 3 years | 1.46 | 1.27 | 1.68 | <0.001 |
| Tertiary, < 3 years | 1.18 | 1.02 | 1.38 | 0.028 |
| Tertiary, ≥ 3 years | Ref. | Ref. | Ref. | Ref. |
| **Prior prescriptions** | 2.84 | 2.58 | 3.13 | <0.001 |
| **Number of comorbidities** | 1.19 | 1.12 | 1.27 | <0.001 |
| **Parental diabetes** | 1.98 | 1.81 | 2.17 | <0.001 |

**Supplementary Figure 1** Data extraction of type 2 diabetes onset date from primary care registers from Scania Region, Region Stockholm, and Västra Götaland Region in addition to national registers to identify new-onset type 2 diabetes within the study period February 1^st^, 2020-December 31^st^, 2022. NDR; National Diabetes Register, T2D; type 2 diabetes

**Supplementary Figure 2** Kaplan-Meier curves comparing COVID-19 exposed with controls in a cohort residing in health care regions Scania, Stockholm or Västra Götaland. Information on type 2 diabetes onset date was gathered from either primary care registers or national registers, whichever date occurred first. Individuals are followed from one day post SARS-CoV-2 infection until type 2 diabetes, emigration, death, or the end of the study period (28 February 2022)

**Supplementary Figure 3** Adjusted hazard ratios (HR) with 95% confidence intervals (95% CI) for the rate of developing type 2 diabetes in a cohort of individuals with confirmed SARS-CoV-2 infection and up to five control individuals matched by sex, age, geographical region, vaccination status, and test date, residing in health care regions Scania, Stockholm or Västra Götaland. Information on type 2 diabetes onset date was gathered from either primary care registers or national registers, whichever date occurred first. The categories for the follow-up period correspond to time since infection. HR and 95% CI were derived using Cox regression adjusting for migration status, number of comorbidities, prior prescriptions, individual disposable income, highest attained education, and parental diabetes.

**Supplementary Figure 4** Kaplan-Meier curves comparing probability of T2D in a cohort of individuals with confirmed SARS-CoV-2 infection and up to five control individuals (unexposed) matched by sex, age, geographical region, vaccination status, and test date, stratified by COVID-19 severity. Individuals are followed from one day post SARS-CoV-2 infection (non-hospitalized) and then transfer to higher severity state (hospitalized or ICU) on day of admission. Individuals are followed until type 2 diabetes, emigration, death, or the end of the study period (28 February 2022)

**Supplementary Figure 5** Kaplan-Meier curves comparing probability of T2D in a cohort of individuals residing in health care regions Scania, Stockholm or Västra Götaland with confirmed SARS-CoV-2 infection and up to five control individuals (unexposed) matched by sex, age, geographical region, vaccination status, and test date, stratified by COVID-19 severity. Information on type 2 diabetes onset date was gathered from either primary care registers or national registers, whichever date occurred first. Individuals are followed from one day post SARS-CoV-2 infection (non-hospitalized) and then transfer to higher severity state (primary care, hospitalized or ICU) on day of admission. Individuals are followed until type 2 diabetes, emigration, death, or the end of the study period (28 February 2022)

**Supplementary Figure 6** Adjusted hazard ratios (HR) with 95% confidence intervals (95% CI) for the rate of developing type 2 diabetes in a cohort of individuals residing in Region Stockholm, Västra Götaland Region, or Scania Region. The cohort include individuals with confirmed SARS-CoV-2 infection and up to five control individuals matched by sex, age, geographical region, vaccination status, and test date, stratified by COVID-19 severity. Information on type 2 diabetes onset date was gathered from either primary care registers or national registers, whichever date occurred first. HR and 95% CI were derived using Cox regression adjusting for migration status, number of comorbidities, prior prescriptions, individual disposable income, highest attained education, and parental diabetes

**Supplementary Figure 7** Kaplan-Meier curves comparing probability of (a) anaemia, (b) chronic kidney disease (CKD), or (c) disorders of the thyroid gland in a cohort of individuals with confirmed SARS-CoV-2 infection and up to five control individuals (unexposed) matched by sex, age, geographical region, vaccination status, and test date, stratified by COVID-19 severity. Individuals are followed from one day post SARS-CoV-2 infection (non-hospitalized) and then transfer to higher severity state (hospitalized or ICU) on day of admission. Individuals are followed until diagnosis of respective negative control, emigration, death, or the end of the study period (28 February 2022)
